# 1290. Impact of Implementing an IV to PO Antibiotic Treatment Protocol for Orthopedic Infections

**DOI:** 10.1093/ofid/ofad500.1129

**Published:** 2023-11-27

**Authors:** Julie Gray, Russell J Benefield, Heather Cummins, Laura Certain

**Affiliations:** University of Utah, Salt Lake City, Utah; University of Utah Health, Salt Lake City, Utah; University of Utah School of Medicine, Salt Lake City, Utah; University of Utah, Salt Lake City, Utah

## Abstract

**Background:**

Oral (PO) regimens have been utilized more frequently for orthopedic infections in place of intravenous (IV) regimens at our center following publication of the OVIVA trial. Given limited published experience of this approach at US medical centers, we reviewed safety and effectiveness outcomes at ours.

**Methods:**

This was a retrospective, propensity-score matched, cohort study of adult patients hospitalized for orthopedic infections from 9/30/2020 to 4/30/2022. Patients discharged on PO antibiotics were matched to patients discharged on IV antibiotics, using a 1:1 nearest neighbor approach without replacement. Variables expected to be associated with treatment selection and/or outcome were included in the propensity score (Table 1). Primary outcomes were early (60-day) and late (1-year) treatment failure following discharge. Secondary outcomes were incidence of adverse drug events (ADE), readmissions, and ED encounters within 60 days of discharge. Outcomes were assessed through chart review.

**Table 1:**
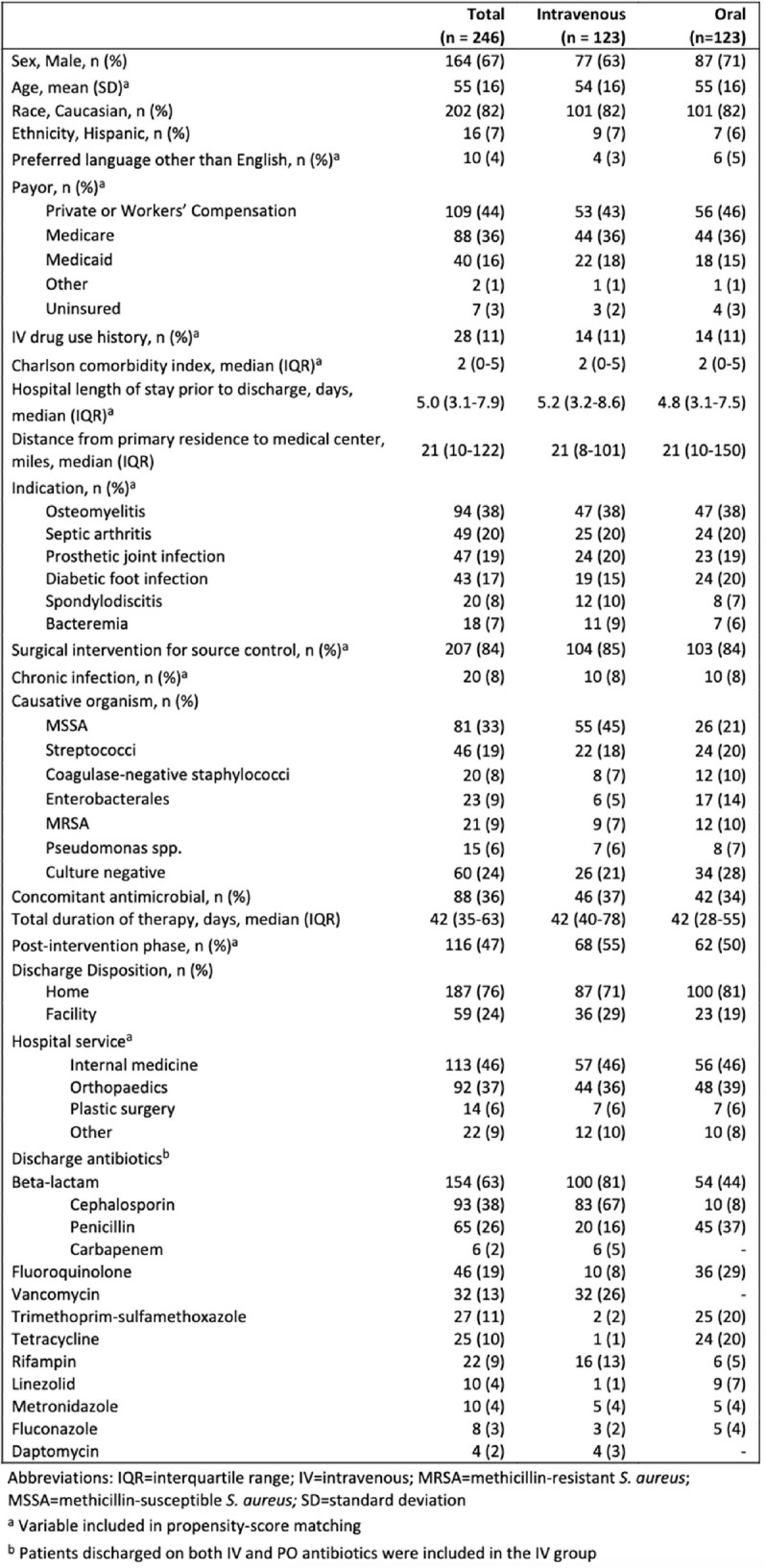
Baseline Characteristics

**Results:**

Six hundred sixty-four patients were screened and 401 met criteria for inclusion. Of these, 123 PO-treated patients were matched to 123 IV-treated patients. Baseline characteristics were similar in the two groups after matching, although there were differences in microbiological indication, and patients discharged on PO regimens were treated for shorter durations and were more likely to be discharged home (Table 1). There was no significant difference in the proportions of patients on PO vs IV antibiotics experiencing treatment failure at 60 days (14% vs 9%, P = 0.23), treatment failure at 1 year (21% vs 20%, P = 0.75), ADE within 60 days (6% vs 11%, P = 0.16), 60-day readmission (26% vs 33%, P = 0.26), or 60-day ED encounter (11% vs 10%, P = 0.83). Survival analyses identified no significant differences in time-to-event between PO and IV treatment for any of the outcomes assessed (Figure 1).

**Figure 1:**
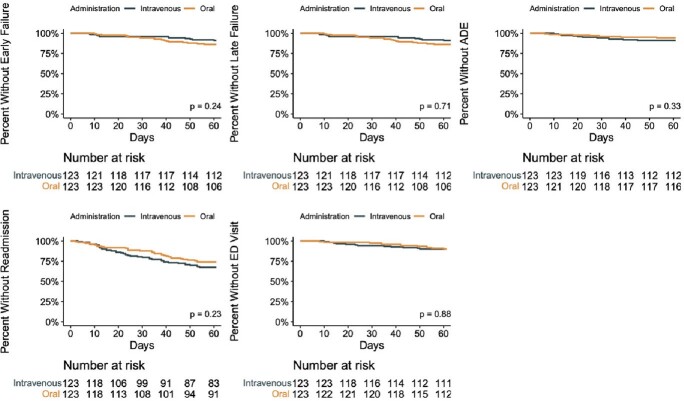
Kaplan-Meier Survival Plots

**Conclusion:**

There was no appreciable difference in outcomes between patients discharged on PO compared to IV regimens. Quality-improvement interventions to increase prescribing of PO antibiotics for the treatment of orthopedic infections should be encouraged.

**Disclosures:**

**Russell J. Benefield, PharmD, BCPS-AQ ID**, Paratek Pharmaceuticals: Grant/Research Support

